# Is There a Link between COVID-19 and Periodontal Disease? A Narrative Review

**DOI:** 10.1055/s-0041-1740223

**Published:** 2022-01-06

**Authors:** Andreas Grigoriadis, Ismo T. Räisänen, Pirjo Pärnänen, Taina Tervahartiala, Timo Sorsa, Dimitra Sakellari

**Affiliations:** 1 Department of Preventive Dentistry, Periodontology and Implant Biology, Dental School, Aristotle University of Thessaloniki, Thessaloniki, Greece; 2 Department of Periodontology, 424 General Military Training Hospital, Thessaloniki, Greece; 3 Department of Oral and Maxillofacial Diseases, Head and Neck Center, University of Helsinki and Helsinki University Hospital, Helsinki, Finland; 4 Department of Dental Medicine, Division of Periodontology, Karolinska Institutet, Stockholm, Sweden

**Keywords:** coronavirus disease 2019, periodontitis, periodontal disease, oral health, oral–general health

## Abstract

The coronavirus disease 2019 (COVID-19) pandemic greatly affected human well-being, social behavior, global economy, and healthcare systems. Everyday clinical practice in dentistry has been adjusted to the increased hazards of aerosol production by routine dental procedures. The objective of this study was to assess the existing literature to determine possible mechanisms of a relationship between COVID-19 and periodontitis, as well as describe findings from relevant epidemiological studies.

Scarce data exist in the literature that directly addresses the relationship between the two diseases. However, several data describe the role of the oral cavity and periodontal tissues as portals of entry of severe acute respiratory syndrome–coronavirus-2 (SARS-CoV-2), and the contribution of cytokines known to be produced in periodontal disease to severe forms of COVID-19. It is also suggested from the current literature that periodontal disease, shown to be associated with systemic diseases such as diabetes mellitus, cardiovascular and respiratory diseases, shares common risk factors with—especially—severe forms of COVID-19.

Further clinical studies are required to establish the relationship between these diseases. Oral hygiene performance and intact periodontal tissues can assist in mitigating the pandemic, and it is suggested that dental practitioners can contribute to identifying at-risk patients.

## Introduction

Severe acute respiratory syndrome–coronavirus-2 (SARS-CoV-2) has caused a pandemic with immense impact on human well-being, social behavior, global economy, and certainly, healthcare systems. As of July 5, 2021, there have been 183,560,151 confirmed cases of COVID-19, including 3,978,581 deaths reported to the World Health Organization (WHO), with the Americas and Europe reporting at least 128 million people affected by the disease. 1

The genome of this novel coronavirus has been identified and fully described in early 2020, triggering a worldwide scientific effort to confront this new challenge. 2 3

COVID-19 presents various clinical signs, with most patients exhibiting mild symptoms including fever, dry cough, anosmia, ageusia, and fatigue. 4 Unfortunately, up to 20% of patients develop severe disease, which leads to hospitalization and possibly necessitates intensive care unit (ICU) treatment, with grave—even fatal—complications, such as acute respiratory distress syndrome (ARDS), multi-organ failures, and sepsis. 4

Despite their similarities regarding structure, pathways of transmission, and clinical symptoms, a higher infectious rate of SARS-CoV-2 compared with SARS-CoV-1 and Middle East Respiratory Syndrome-CoV (MERS–CoV) has been reported, contributing to the global spread and raising substantial concerns for controlling the transmission of the infection. 5 6

Transmission of SARS-CoV-2 occurs mainly via respiratory droplets expelled by talking, sneezing, coughing from face-to-face contact and, to a lesser degree, via contaminated surfaces, and it is estimated that up to 59% of all transmissions came from the asymptomatic transmission, comprising 35% from presymptomatic subjects, and 24% from subjects who never develop symptoms. 7

Of particular concern for the dental community is that aerosol spread of the virus can occur. 8 Several dental procedures in clinical practice generate aerosol, and the close contact of the dental practitioner with the patient leads to an increased risk of transmission. Consequently, the implementation of routine dental procedures has been affected worldwide, with aerosol-generating procedures generally suspended, practice limited to emergency cases, and the introduction of teledentistry—when feasible. 9 10

One year after the onset of the pandemic, various treatments have been proposed –and tested for the disease, mostly under pressure for treating patients at risk for severe clinical symptomatology. 11

A great hope for mitigation of the pandemic has been the development of safe and effective vaccines against SARS-CoV-2. Due to the collaboration of the scientific community, pharmaceutical companies, and health organizations globally, certain vaccines are already authorized for human use, with a substantial number under clinical trials. 12 As of July 5, 2021, it is estimated that 2,988,941,529 vaccine doses have been administered. 1

Of particular interest for the dental community is the possible relationship between COVID-19 and periodontal disease.

Therefore, the purpose of the present narrative review was to discuss scientific information on the role of the oral cavity and periodontal tissues as portals of entry of SARS-CoV-2, the connection of risk factors for COVID-19 with periodontal disease, and the biological plausibility of this relationship.

## Methods

A comprehensive search was performed in three electronic databases (MEDLINE/PubMed, Scopus, Cochrane Controlled register of Trials) from January 2020 until February 2021. Manual searching was also performed on Google and Google Scholar. Only articles written in English were considered initially. The following keywords were used in the search: “periodontal disease,” “COVID-19,” “SARS-CoV-2,” “oral cavity,” “oral hygiene,” “ACE2 receptors,” “periodontitis risk factors,” and ”COVID-19 risk factors.'' All types of articles considered relevant for discussion were selected and analyzed in detail. The reference lists of the selected articles were then hand-searched for additional articles deemed appropriate by two of the authors (AG and DS). The literature search located 193 studies. After duplicate removal, 81 studies were assessed based on the title and abstract. Of them, 66 studies were examined as full-text articles. Twenty-seven articles and information from official Web sites describing the epidemiology, transmission, pathophysiology, and treatment of COVID-19 were also included in the present review.

### Interrelation between SARS-CoV-2 and the Oral Cavity

Of pivotal importance for the comprehension of SARS-CoV-2 pathogenesis was recognizing the specific receptors utilized by the virus to invade host cells. It has been shown that the Angiotensin-Converting Enzyme -2 (ACE-2) consists of a major route for viral entry to the cells, similarly to SARS-CoV-1. 13 14 In addition, it was reported that the affinity of binding of the viral spike (S) protein is 10 to 20 fold increased compared with SARS-CoV-1, and this fact could explain the highly contagious ability of this specific virus. 14 15 ACE2 is not the only molecule promoting viral invasiveness. Transmembrane protease serine 2 (TMPRSS2) and furin are also described as cell entry assisting molecules for SARS-CoV-2, albeit to a lesser extent. 13 ACE-2 is distributed throughout several tissues, and its' expression and function are affected by several factors, including genetic and demographic characteristics, lifestyle habits, comorbidities, and concurrent medications. 14 It is already known that ACE2 is highly expressed on epithelial cells of principal target organs of SARS-CoV-2 such as the lungs, kidneys, and intestines. 14 16

### These Receptors are Expressed in the Oral Cavity

According to the report of Xu et al, ACE2 is expressed on the epithelial cells of various areas of the oral mucosa, particularly on the tongue compared with buccal or gingival cells, thus implicating the oral cavity as a highly susceptible environment for SARS-CoV-2 infection. 6 These findings were further enhanced by consequent studies, applying a variety of techniques, in which it has been shown that enriched ACE2 expression is observed apart from the surface of the tongue in several areas of the head and neck region. Examples of those areas are the oral cavity epithelial cells (including the gingival sulcus/periodontal pocket epithelium), the salivary glands, the vocal cords, and the sinuses. 17 18 19

ACE2 is also identified in patients with periodontal disease in various cell types, such as fibroblasts, osteoblasts, and osteoclasts, which are critical for homeostasis for hard and soft tissues around teeth and implants. 20 21 Earlier studies in animals have also demonstrated ACE2 expression in gingival fibroblasts. 22 Therefore, it is suggested that this line of research will elucidate methods of viral transmission leading to preventive strategies and explain aspects of the pathophysiology of COVID-19. For example, the ACE2 (TMPRSS2) and abundant furin expression on taste bud-derived cells cultures might explain the olfactory abnormality, one of the known causes of dysgeusia in COVID-19. 17 Based on these findings, several authors have formulated a hypothesis that the oral environment, including gingival sulci/periodontal pockets 23 24 and salivary glands, 25 can serve as potential reservoirs for shedding SARS-CoV-2. Epithelial cells of salivary glands have previously been shown to be infected in studies regarding SARS-CoV-1 and were considered a major source for the virus in saliva, 26 and the same may apply for SARS-CoV-2. The presence of increased viral load in saliva samples and the recently reported detection of viral RNA in gingival crevicular fluid (GCF) also demonstrate the connection between COVID-19 disease and the oral cavity. 27 28 29 30 Due to this fact, saliva sampling and analysis for detecting SARS-CoV-2 infection have been supported by several studies. In addition, saliva sampling and analysis offer ease convenience of patients and non-invasiveness and could serve as a diagnostic tool for large-scale screening, compared with the “gold-standard,” the nasopharyngeal swab nuclear acid amplification testing (NAAT). Nevertheless, it should be noticed that findings in the literature regarding the diagnostic value of saliva sampling have not been unequivocal or provide similar performance to the gold standard. 28 31 32 Results from a recently published systematic review and meta-analysis suggest that saliva NAAT diagnostic accuracy is similar to nasopharyngeal swab NAAT, especially in the ambulatory setting, suggesting larger-scale research on its' alternative use. 33 In line with these findings, the Federal Drug Administration (FDA) has authorized SARS-CoV-2 testing on saliva samples collected at home using a self-collection kit in the United States Federal Drug Administration. 34 Findings from a May 2021 study investigating SARS-CoV-2 infection of the oral cavity and saliva have strongly confirmed that the oral cavity is an important site for infection from the virus and that saliva is suggested as a route for transmission. 35

Although the data mentioned above describe the oral environment as biologically favorable for SARS-CoV-2 invasion, proliferation, and shedding source, one should keep in mind that it also consists of an effective barrier against microbial pathogens. Effective innate immunity mechanisms and locally produced molecules such as the defensins constitute a well-documented first line of defense that might not allow for colonization and proliferation of the virus. These properties may account for controversial results reported in the literature. 36 37

### Risk Factors for COVID-19 and Periodontal Disease

Several risk factors have emerged following the surge and spread of COVID-19, especially for cases with severe complications and are still under investigation. According to the Centers for Disease Control (USA), the following underlying medical conditions consist risk factors for developing severe COVID-19: cancer, chronic kidney disease, chronic obstructive pulmonary disease (COPD), Down's syndrome, heart conditions, such as heart failure, coronary artery disease or cardiomyopathies, solid organ transplants, obesity (body mass index of 30 kg/m ^2^ or higher), pregnancy, sickle cell disease, smoking, and type 2 diabetes. 38

Among them, certain conditions and clinical entities are strongly related to periodontal disease. Comorbidities, as reported so far for COVID-19, are also known to affect the onset, extent, and severity of periodontal diseases, and this relationship is worthy of investigating since it could positively affect the recognition and early intervention of at-risk individuals.

For example, age > 65 years is considered a significant risk factor for severe COVID-19, probably due to comorbidities and the reduced immune response in this age group, and it is also suggested that male subjects are also more prone to complications of the disease. 39 Age is an established risk factor for periodontal disease, 40 providing a link for dental practitioners to emphasize the need for optimal oral hygiene and healthy periodontal tissues to older patients to reduce complications of both disease entities similarly to other chronic diseases.

Obesity is also reported to increase the risk for severe COVID-19 disease, probably due to reduced respiratory function and amplified inflammatory response. The presence of a “primed” inflammatory status with increased production of pro-inflammatory cytokines and reactive oxygen species in obese individuals has also been suggested as a risk factor for the development of periodontal disease. 41

This is also the case for the link between cardiovascular and periodontal disease, a relationship widely documented in the literature at the biological and epidemiological levels. 42 Comorbidities such as heart failure, coronary artery disease, cardiomyopathies, and pulmonary hypertension are currently considered risk factors for severe COVID-19 disease. In contrast, other cardiovascular or cerebrovascular diseases, such as hypertension or stroke, might increase the risk of severe illness from the disease. 38

A plausible mechanism of the interplay between periodontitis, the above-mentioned cardiovascular conditions, and COVID-19, is the increase of systemic inflammation induced by diseased periodontal tissues with over-production of pro-inflammatory mediators such as cytokines, both locally and systemically. A “cytokine storm,” as described below, is a common finding in severe COVID-19 infections, suggesting a possible exacerbation when periodontal disease is present in the oral cavity.

Chronic obstructive pulmonary disease (COPD) refers to a group of diseases that cause airflow blockage and breathing-related problems, including emphysema and chronic bronchitis. The association between periodontitis and respiratory diseases (asthma, COPD, and pneumonia) has a strong biological background based on the potential role of oral bacteria to facilitate infection from respiratory pathogens. This is also strongly suggested by epidemiological findings. 43 44 Recent studies have shown that severe periodontal disease increases the risk for COPD mortality in older patients, and on the other hand, in subjects over 70 years, enhanced oral hygiene can prevent airway infections. 45 46 Given the strong relation of severe COVID-19 to COPD, dental practitioners could thus be helpful to identify risk groups among patients exhibiting COPD.

The bidirectional linking of periodontal disease and diabetes is well established in the literature. 47 Data from recent large-scale clinical studies have shown that risks of fatal or intensive care unit treated COVID-19 cases were elevated in patients with diabetes mellitus types 1 and 2, or even admission hyperglycemia regardless of prior diagnosis of diabetes. 48 49 Hyperglycemia is known to dysregulate the immune response, promote the infection of cells from SARS-CoV-2 by glycosylation of ACE2, and elevate the gene expression of matrix-metalloproteinases which promote inflammation.

## Biological Plausibility and Review of the Literature

The SARS-CoV-2 infection triggers a profound cytokine response in the host, often referred to as a 'cytokine storm' comprising a series of mediators important for inflammation but not assisting ineffective virus clearance. 50

This 'cytokine storm' is exhibited as elevated serum levels of TNF-α, IL-1β, IL-6, IL-8, G-CSF and GM-CSF, and chemokines, such as MCP1, IP10, and MIP1α, with higher levels in those who are critically ill. Elevated Th17 pathway responses could lead to severe pulmonary edema and tissue damage in lung infections and have also been observed in patients of SARS-CoV-1 and MERS-CoV. 50 51 52 53

It is known that targeting the cytokines mentioned above with specific antibodies such as sarilumab and tocilizumab is an effective pharmacological approach for immune-mediated inflammatory diseases (IMIDs), such as rheumatoid arthritis (RA), spondyloarthritis (SpA), and Crohn's disease. 11 The inhibition of cytokines might be considered inducing immune suppression, but these molecules are known to neutralize specific mediators, therefore attenuating the hyperinflammatory reaction to the SARS-CoV-2 infection and not inducing a generalized and harmful immune suppression. 50 Therefore, monoclonal antibodies directed against key inflammatory cytokines consist of a potential class of adjunctive therapies for COVID-19 under investigation. 11

Of interest is the fact that ACE2 functions as a down-regulator of the inflammatory response by blocking oxidative stress, cell proliferation, and the release of pro-inflammatory mediators. In COVID-19 patients, a reduction of ACE2 levels is observed due to the complexes formed between the virus and these proteins, and this fact might also contribute to the “cytokine storm” observed, especially in severe cases of the disease. 54

Similarly, immune-mediated pathogenetic pathways have also been shown for periodontitis, and the cytokines produced locally in the periodontal tissues are implicated in the interaction mechanism of periodontal inflammation and systemic diseases. 55 This fact supports the hypothesis that meticulous oral hygiene and intact, non-inflamed periodontal tissues can prevent COVID-19 complications, at least in certain patient groups.

Matrix metalloproteinases (MMPs) are involved in tissue destructive oral diseases such as periodontitis, peri-implantitis, dental caries, and oral cancer. 56 MMP-8, also known as neutrophil collagenase, causes unwanted tissue destruction in periodontitis/peri-implantitis due to increased activation of MMP-8 inactive periodontitis/peri-implantitis, 57 which has also been demonstrated to be reflected in oral fluids in vivo. 58 Furthermore, previous studies have shown an association between elevated levels of active MMP-8 (aMMP-8) in oral fluids and clinical periodontal parameters. 59 60 61 62 In addition, diabetes is known to elevate and activate MMPs in gingival tissues, and oral fluids and recent studies have shown the usefulness of aMMP-8 point-of-care testing in the clinic concerning the identification of patients at risk of prediabetes, gestational diabetes, and progression of periodontitis in HNC radiotherapy. 63 64 65 66 Given the ability of the test to conveniently identify people with diabetes with periodontitis, the possibility to identify COVID-risk patients such as the populations mentioned above is eventually exerted.

The relationship between periodontitis and systemic diseases and the link between them and the active form of periodontitis, reflected as elevated aMMP-8 levels in oral fluids, makes the aMMP-8 point-of-care testing an interesting tool for future COVID-19 studies. 67

The possible coexistence of COVID-19 and periodontitis and the impact of oral hygiene could be a critical situation to observe to minimize severe manifestation of the disease and assist in identifying people at risk in the dental office.

In fact, according to the existing literature, several authors have, so far, underlined this possible interaction, the biological plausibility, and mechanisms and implications for clinical practice of the interplay between COVID-19 and periodontitis. 68 69 70 71 72 73

Scarce data from clinical trials in the current literature exist regarding the impact of periodontal disease in severe COVID-19 cases. In a study with more than 13.000 participants in the United Kingdom, 12% were COVID-19 positive, self-reported painful or bleeding gums, and loose teeth were used as surrogate markers for periodontal disease. 74 According to the study's findings, the risk of COVID-19 infection in participants with painful or bleeding gums and loose teeth compared with controls was not increased, while COVID-19 positive participants with painful or bleeding gums had a higher risk of mortality but not hospital admission. No correlation was found between self-reported loss of teeth and higher risk of hospital admission or mortality than the control group. Although evidence from this study is inconclusive, it should be emphasized that, according to findings, amongst the COVID-19 positive, there was significantly higher mortality for participants with periodontal disease. This line of research with clinical assessments of periodontal disease and the combination of dental and medical records, especially in hospital settings, will certainly improve our current understanding of the interplay between these diseases.

A recently published case-control study, involving 568 patients in Qatar diagnosed with COVID-19, has shown that periodontitis was associated with 3.5 times higher risk of admission to ICU, 4.5 times need for mechanical ventilation, and almost nine times for death in Covid-19 patients, after adjusting for age, sex, BMI, smoking status, and other conditions. These findings were based on electronic medical and dental data and, albeit the limitations of the study (it involved only diagnostic radiographic criteria and was not designed to address causality), it is suggested that establishing and maintaining periodontal health may become an important part of the care of these patients. 75

It has also been suggested that the relationship between the oral microbiome and severe COVID-19 infection should be investigated to enlighten the outcomes of COVID-19 disease. The main comorbidities associated with an increased risk of severe, even fatal from COVID-19 as described above, are also associated with altered oral biofilms and periodontal disease. Hence, the link between poor oral health and COVID-19 complications is suggested. 76 Future studies aiming at defining the microbiome in patients with both clinical entities could clarify the impact of periodontitis on COVID-19 infection and the clinical course of the disease. In the context of the pandemic, until the importance of oral hygiene and the presence of oral disease in the severity and mortality risk of COVID-19 is solidly proven, the maintenance and optimization of oral hygiene should be emphasized to dental patients to target the reduction of the viral load in the oral cavity. In the same context, in anticipation of well-designed pre-clinical and clinical trials, the adjunctive use of various mouth rinses (mainly cetylpyridinium chloride, povidone–iodine, or chlorhexidine and hydrogen peroxide) has also been suggested, both as single preprocedural use and as daily use during a limited period. 77

## Conclusions

The surge of the COVID-19 pandemic is a challenge for periodontology. Exploring the mechanisms of interplay between COVID-19 and periodontal disease can contribute to the identification of patients at risk for complications and mitigate the spread and symptoms of COVID-19 by controlling periodontal inflammation and improving oral hygiene and health.

## References

[1] COVID-19 situation in the WHO European RegionAvailable at:https://who.maps.arcgis.com/apps/dashboards/ead3c6475654481ca51c248d52ab9c61

[2] Lu R Zhao X Li J Genomic characterisation and epidemiology of 2019 novel coronavirus: implications for virus origins and receptor binding Lancet 2020 395(10224):565 574 32007145 10.1016/S0140-6736(20)30251-8 PMC7159086

[3] Zhang Y-Z Holmes E C A genomic perspective on the origin and emergence of SARS-CoV-2 Cell 2020 181 02 223 227 32220310 10.1016/j.cell.2020.03.035 PMC7194821

[4] Wiersinga W J Rhodes A Cheng A C Peacock S J Prescott H C Pathophysiology, transmission, diagnosis, and treatment of coronavirus disease 2019 (COVID-19): a review JAMA 2020 324 08 782 793 32648899 10.1001/jama.2020.12839

[5] Abdelrahman Z Li M Wang X Comparative review of SARS-CoV-2, SARS-CoV, MERS-CoV, and influenza a respiratory viruses Front Immunol 2020 11 552909 33013925 10.3389/fimmu.2020.552909 PMC7516028

[6] Hu T Liu Y Zhao M Zhuang Q Xu L He Q A comparison of COVID-19, SARS and MERS PeerJ 2020 8 e9725 32879801 10.7717/peerj.9725 PMC7443081

[7] Johansson M A Quandelacy T M Kada S SARS-CoV-2 transmission from people without COVID-19 symptoms JAMA Netw Open 2021 4 01 e2035057 e2035057 33410879 10.1001/jamanetworkopen.2020.35057 PMC7791354

[8] Peng X Xu X Li Y Cheng L Zhou X Ren B Transmission routes of 2019-nCoV and controls in dental practice Int J Oral Sci 2020 12 01 9 32127517 10.1038/s41368-020-0075-9 PMC7054527

[9] Guidance for dental settingsAccessed Nov 8, 2021 at.https://www.cdc.gov/coronavirus/2019-ncov/hcp/dental-settings.html

[10] EFP suggestions for the management of a dental clinic during the Covid-19 pandemicAccessed Nov 8, 2021 at:https://www.efp.org/fileadmin/uploads/efp/Documents/covid19SafetyProtocol.pdf

[11] Sanders J M Monogue M L Jodlowski T Z Cutrell J B Pharmacologic treatments for coronavirus disease 2019 (COVID-19): a review JAMA 2020 323 18 1824 1836 32282022 10.1001/jama.2020.6019

[12] COVID-19 vaccinesAccessed Nov 8, 2021 at:https://www.who.int/emergencies/diseases/novel-coronavirus-2019/covid-19-vaccines

[13] Hoffmann M Kleine-Weber H Schroeder S SARS-CoV-2 cell entry depends on ACE2 and TMPRSS2 and is blocked by a clinically proven protease inhibitor Cell 2020 181 02 271 2.8E10 32142651 10.1016/j.cell.2020.02.052 PMC7102627

[14] Bourgonje A R Abdulle A E Timens W Angiotensin-converting enzyme 2 (ACE2), SARS-CoV-2 and the pathophysiology of coronavirus disease 2019 (COVID-19) J Pathol 2020 251 03 228 248 32418199 10.1002/path.5471 PMC7276767

[15] Wrapp D Wang N Corbett K S Cryo-EM structure of the 2019-nCoV spike in the prefusion conformation Science 2020 367(6483):1260 1263 32075877 10.1126/science.abb2507 PMC7164637

[16] Hamming I Timens W Bulthuis M L Lely A T Navis G van Goor H Tissue distribution of ACE2 protein, the functional receptor for SARS coronavirus. A first step in understanding SARS pathogenesis J Pathol 2004 203 02 631 637 15141377 10.1002/path.1570 PMC7167720

[17] Sakaguchi W Kubota N Shimizu T Existence of SARS-CoV-2 entry molecules in the oral cavity Int J Mol Sci 2020 21 17 6000 10.3390/ijms21176000 PMC7503451 32825469

[18] Descamps G Verset L Trelcat A ACE2 protein landscape in the head and neck region: the conundrum of SARS-CoV-2 infection Biology (Basel) 2020 9 08 235 10.3390/biology9080235 PMC7465650 32824830

[19] Wu C Zheng M Single-cell RNA expression profiling shows that ACE2, the putative receptor of COVID-2019, has significant expression in nasal and mouth tissue, and is co-expressed with TMPRSS2 and not co-expressed with SLC6A19 in the tissues Preprints 2020 10.21203/rs.3.rs-16992/v1

[20] Gabriele L G Morandini A C Dionísio T J Santos C F Angiotensin II type 1 receptor knockdown impairs interleukin-1β-induced cytokines in human periodontal fibroblasts J Periodontol 2017 88 01 e1 e11 27541080 10.1902/jop.2016.160354

[21] Santos C F Morandini A C Dionísio T J Functional local renin-angiotensin system in human and rat periodontal tissue PLoS One 2015 10 08 e0134601 26244896 10.1371/journal.pone.0134601 PMC4526652

[22] Santos C F Akashi A E Dionísio T J Characterization of a local renin-angiotensin system in rat gingival tissue J Periodontol 2009 80 01 130 139 19228099 10.1902/jop.2009.080264 PMC2758819

[23] Badran Z Gaudin A Struillou X Amador G Soueidan A Periodontal pockets: a potential reservoir for SARS-CoV-2? Med Hypotheses 2020 143 109907 32504927 10.1016/j.mehy.2020.109907 PMC7833827

[24] Kheur S Kheur M Gupta A A Raj A T Is the gingival sulcus a potential niche for SARS-corona virus-2? Med Hypotheses 2020 143 109892 32498008 10.1016/j.mehy.2020.109892 PMC7255245

[25] Xu J Li Y Gan F Du Y Yao Y Salivary glands: potential reservoirs for COVID-19 asymptomatic infection J Dent Res 2020 99 08 989 989 32271653 10.1177/0022034520918518

[26] Liu L Wei Q Alvarez X Epithelial cells lining salivary gland ducts are early target cells of severe acute respiratory syndrome coronavirus infection in the upper respiratory tracts of rhesus macaques J Virol 2011 85 08 4025 4030 21289121 10.1128/JVI.02292-10 PMC3126125

[27] Wyllie A L Fournier J Casanovas-Massana A Saliva or nasopharyngeal swab specimens for detection of SARS-CoV-2 N Engl J Med 2020 383 13 1283 1286 32857487 10.1056/NEJMc2016359 PMC7484747

[28] Gupta S Mohindra R Chauhan P SARS-CoV-2 detection in gingival crevicular fluid J Dent Res 2021 100 02 187 193 33138663 10.1177/0022034520970536 PMC7642823

[29] To K KW Yip C CY Lai C YW Saliva as a diagnostic specimen for testing respiratory virus by a point-of-care molecular assay: a diagnostic validity study Clin Microbiol Infect 2019 25 03 372 378 29906597 10.1016/j.cmi.2018.06.009

[30] Zhu J Guo J Xu Y Chen X Viral dynamics of SARS-CoV-2 in saliva from infected patients J Infect 2020 81 03 e48 e50 32593658 10.1016/j.jinf.2020.06.059 PMC7316041

[31] Jamal A J Mohammad M Coomes E Sensitivity of nasopharyngeal swabs and saliva for the detection of severe acute respiratory syndrome coronavirus 2 (SARS-CoV-2) medRxiv 2020 10.1101/2020.05.01.20081026 PMC7337630 32584972

[32] Pasomsub E Watcharananan S P Boonyawat K Saliva sample as a non-invasive specimen for the diagnosis of coronavirus disease 2019: a cross-sectional study Clin Microbiol Infect 2021 27 02 2850 2.85E6 10.1016/j.cmi.2020.05.001 PMC7227531 32422408

[33] Butler-Laporte G Lawandi A Schiller I Comparison of Saliva and Nasopharyngeal Swab Nucleic Acid Amplification Testing for Detection of SARS-CoV-2: A Systematic Review and Meta-analysisJAMA Intern Med. 10.1001/jamainternmed.2020.8876 PMC7811189 33449069

[34] Coronavirus (COVID-19) Update: FDA Authorizes First Diagnostic Test Using At-Home Collection of Saliva SpecimensAccessed Nov 8, 2021 at:https://www.fda.gov/news-events/press-announcements/coronavirus-covid-19-update-fda-authorizes-first-diagnostic-test-using-home-collection-saliva

[35] NIH COVID-19 Autopsy Consortium HCA Oral and Craniofacial Biological Network Huang N Pérez P Kato T SARS-CoV-2 infection of the oral cavity and saliva Nat Med 2021 27 05 892 903 33767405 10.1038/s41591-021-01296-8 PMC8240394

[36] Troeltzsch M Berndt R Troeltzsch M Is the oral cavity a reservoir for prolonged SARS-CoV-2 shedding? Med Hypotheses 2021 146 110419 33309251 10.1016/j.mehy.2020.110419 PMC7683957

[37] Fernandes Matuck B Dolhnikoff M Maia G VA Periodontal tissues are targets for Sars-Cov-2: a post-mortem study J Oral Microbiol 2020 13 01 1.848135E6 10.1080/20002297.2020.1848135 PMC7717160 33391625

[38] People with Certain Medical ConditionsAccessed Nov 8, 2021 at.https://www.cdc.gov/coronavirus/2019-ncov/need-extra-precautions/people-with-medical-conditions.html

[39] Jordan R E Adab P Cheng K COVID-19: risk factors for severe disease and death BMJ 2020 10.1136/bmj.m1198 32217618

[40] Persson G R Periodontal complications with age Periodontol 2000 2018 78 01 185 194 30198125 10.1111/prd.12227

[41] Suvan J Harrington Z Petrie A Obesity as predictive factor of periodontal therapy clinical outcomes: a cohort study J Clin Periodontol 2020 47 05 594 601 31994205 10.1111/jcpe.13261

[42] Sanz M Marco Del Castillo A Jepsen S Periodontitis and cardiovascular diseases: Consensus report J Clin Periodontol 2020 47 03 268 288 32011025 10.1111/jcpe.13189 PMC7027895

[43] Gomes-Filho I S Cruz S SD Trindade S C Periodontitis and respiratory diseases: A systematic review with meta-analysis Oral Dis 2020 26 02 439 446 31715080 10.1111/odi.13228

[44] Huffnagle G B Dickson R P Lukacs N W The respiratory tract microbiome and lung inflammation: a two-way street Mucosal Immunol 2017 10 02 299 306 27966551 10.1038/mi.2016.108 PMC5765541

[45] Sjögren P Nilsson E Forsell M Johansson O Hoogstraate J A systematic review of the preventive effect of oral hygiene on pneumonia and respiratory tract infection in elderly people in hospitals and nursing homes: effect estimates and methodological quality of randomized controlled trials J Am Geriatr Soc 2008 56 11 2124 2130 18795989 10.1111/j.1532-5415.2008.01926.x

[46] Qian Y Yuan W Mei N Periodontitis increases the risk of respiratory disease mortality in older patients Exp Gerontol 2020 133 110878 32061644 10.1016/j.exger.2020.110878

[47] Sanz M Ceriello A Buysschaert M Scientific evidence on the links between periodontal diseases and diabetes: consensus report and guidelines of the joint workshop on periodontal diseases and diabetes by the International Diabetes Federation and the European Federation of Periodontology J Clin Periodontol 2018 45 02 138 149 29280174 10.1111/jcpe.12808

[48] SEMI-COVID-19 Network Carrasco-Sánchez F J López-Carmona M D Martínez-Marcos F J Admission hyperglycaemia as a predictor of mortality in patients hospitalized with COVID-19 regardless of diabetes status: data from the Spanish SEMI-COVID-19 Registry Ann Med 2021 53 01 103 116 33063540 10.1080/07853890.2020.1836566 PMC7651248

[49] Public Health Scotland COVID-19 Health Protection Study Group Scottish Diabetes Research Network Epidemiology Group McGurnaghan S J Weir A Bishop J Risks of and risk factors for COVID-19 disease in people with diabetes: a cohort study of the total population of Scotland Lancet Diabetes Endocrinol 2021 9 02 82 93 33357491 10.1016/S2213-8587(20)30405-8 PMC7832778

[50] Schett G Sticherling M Neurath M F COVID-19: risk for cytokine targeting in chronic inflammatory diseases? Nat Rev Immunol 2020 20 05 271 272 32296135 10.1038/s41577-020-0312-7 PMC7186927

[51] Wu D Yang X O TH17 responses in cytokine storm of COVID-19: An emerging target of JAK2 inhibitor Fedratinib J Microbiol Immunol Infect 2020 53 03 368 370 32205092 10.1016/j.jmii.2020.03.005 PMC7156211

[52] Sahni V Gupta S COVID-19 & Periodontitis: the cytokine connection Med Hypotheses 2020 144 109908 32534336 10.1016/j.mehy.2020.109908 PMC7832148

[53] Fabri G MC Potential Link between COVID-19 and Periodontitis: Cytokine Storm, Immunosuppression, and Dysbiosis Oral Health Dent Manag 2020 20 1 5

[54] Mancini L Quinzi V Mummolo S Marzo G Marchetti E Angiotensin-converting enzyme 2 as a possible correlation between COVID-19 and periodontal Disease Appl Sci (Basel) 2020 10 6224

[55] Cekici A Kantarci A Hasturk H Van Dyke T E Inflammatory and immune pathways in the pathogenesis of periodontal disease Periodontol 2000 2014 64 01 57 80 24320956 10.1111/prd.12002 PMC4500791

[56] Sorsa T Tjäderhane L Salo T Matrix metalloproteinases (MMPs) in oral diseases Oral Dis 2004 10 06 311 318 15533204 10.1111/j.1601-0825.2004.01038.x

[57] Sorsa T Tjäderhane L Konttinen Y T Matrix metalloproteinases: contribution to pathogenesis, diagnosis and treatment of periodontal inflammation Ann Med 2006 38 05 306 321 16938801 10.1080/07853890600800103

[58] Lee W Aitken S Sodek J McCulloch C A Evidence of a direct relationship between neutrophil collagenase activity and periodontal tissue destruction in vivo: role of active enzyme in human periodontitis J Periodontal Res 1995 30 01 23 33 7722844 10.1111/j.1600-0765.1995.tb01249.x

[59] Sorsa T Ingman T Suomalainen K Cellular source and tetracycline-inhibition of gingival crevicular fluid collagenase of patients with labile diabetes mellitus J Clin Periodontol 1992 19 02 146 149 1318330 10.1111/j.1600-051x.1992.tb00454.x

[60] Ryan M E Ramamurthy N S Sorsa T Golub L M MMP-mediated events in diabetes Ann N Y Acad Sci 1999 878 311 334 10415738 10.1111/j.1749-6632.1999.tb07692.x

[61] Safkan-Seppälä B Sorsa T Tervahartiala T Beklen A Konttinen Y T Collagenases in gingival crevicular fluid in type 1 diabetes mellitus J Periodontol 2006 77 02 189 194 16460243 10.1902/jop.2006.040322

[62] Rathnayake N Åkerman S Klinge B Salivary biomarkers of oral health: a cross-sectional study J Clin Periodontol 2013 40 02 140 147 23174014 10.1111/jcpe.12038

[63] Grigoriadis A Sorsa T Räisänen I Pärnänen P Tervahartiala T Sakellari D Prediabetes/diabetes can be screened at the dental office by a low-cost and fast chair-side/point-of-care aMMP-8 immunotest Diagnostics (Basel) 2019 9 04 151 10.3390/diagnostics9040151 PMC6963402 31627410

[64] Grigoriadis A Räisänen I T Pärnänen P Tervahartiala T Sorsa T Sakellari D Prediabetes/diabetes screening strategy at the periodontal clinic Clin Exp Dent Res 2021 7 01 85 92 33300692 10.1002/cre2.338 PMC7853879

[65] Chaparro A Realini O Hernández M Early pregnancy levels of gingival crevicular fluid matrix metalloproteinases-8 and− 9 are associated with the severity of periodontitis and the development of gestational diabetes mellitus J Periodontol 2021 92 02 205 215 32789908 10.1002/JPER.19-0743

[66] Keskin M Lähteenmäki H Rathnayake N Active matrix metalloproteinase-8 and interleukin-6 detect periodontal degeneration caused by radiotherapy of head and neck cancer: a pilot study Expert Rev Proteomic 2021 17 10 777 784 10.1080/14789450.2020.1858056 33406924

[67] Räisänen I T Umeizudike K A Pärnänen P Periodontal disease and targeted prevention using aMMP-8 point-of-care oral fluid analytics in the COVID-19 era Med Hypotheses 2020 144 110276 33254580 10.1016/j.mehy.2020.110276 PMC7492808

[68] Bertolini M Pita A Koo S Cardenas A Methil A Periodontal disease in the COVID-19 era: potential reservoir and increased risk for SARS-CoV-2 Pesqui Bras Odontopediatria Clin Integr 2020 20(supp1):e0134 https://doi.org/10.1590/pboci.2020.162

[69] Sharma A Chauhan S Sharma R Jindal V Rajput P Jindal V How chronic periodontitis associated with systemic diseases increases the case fatality rate among COVID-19 Patients J Adv Med Dent Scie Res 2020 8 71 77

[70] Pitones-Rubio V Chávez-Cortez E G Hurtado-Camarena A González-Rascón A Serafín-Higuera N Is periodontal disease a risk factor for severe COVID-19 illness? Med Hypotheses 2020 144 109969 32592918 10.1016/j.mehy.2020.109969 PMC7303044

[71] Siddharthan S Naing N N Wan-Arfah N Periodontal disease and COVID 19 J Pharm Res Int 2020 32 32 88 91 10.9734/JPRI/2020/v32i3230937

[72] Kadkhodazadeh M Amid R Moscowchi A Does COVID-19 affect periodontal and peri-implant diseases? J Long Term Eff Med Implants 2020 30 01 1 2 33389910 10.1615/JLongTermEffMedImplants.2020034882

[73] Madapusi Balaji T Varadarajan S Rao U SV Oral cancer and periodontal disease increase the risk of COVID 19? A mechanism mediated through furin and cathepsin overexpression Med Hypotheses 2020 144 109936 32505073 10.1016/j.mehy.2020.109936 PMC7263251

[74] Larvin H Wilmott S Wu J Kang J The impact of periodontal disease on hospital admission and mortality during COVID-19 pandemic Front Med (Lausanne) 2020 7 604980 33330570 10.3389/fmed.2020.604980 PMC7719810

[75] Marouf N Cai W Said K N Association between periodontitis and severity of COVID-19 infection: a case-control study J Clin Periodontol 2021 48 04 483 491 33527378 10.1111/jcpe.13435 PMC8014679

[76] Sampson V Kamona N Sampson A Could there be a link between oral hygiene and the severity of SARS-CoV-2 infections? Br Dent J 2020 228 12 971 975 32591714 10.1038/s41415-020-1747-8 PMC7319209

[77] Herrera D Serrano J Roldán S Sanz M Is the oral cavity relevant in SARS-CoV-2 pandemic? Clin Oral Investig 2020 24 08 2925 2930 10.1007/s00784-020-03413-2 PMC7309196 32577830

